# A Preliminary Analysis of the Immunoglobulin Genes in the African Elephant (*Loxodonta africana*)

**DOI:** 10.1371/journal.pone.0016889

**Published:** 2011-02-25

**Authors:** Yongchen Guo, Yonghua Bao, Hui Wang, Xiaoxiang Hu, Zhihui Zhao, Ning Li, Yaofeng Zhao

**Affiliations:** 1 State Key Laboratory of AgroBiotechnology, College of Biological Sciences, China Agricultural University, Beijing, People's Republic of China; 2 Department of Basic Immunology, Xinxiang Medical University, Xinxiang, People's Republic of China; 3 Agricultural Division, College of Animal Science and Veterinary Medicine, Jilin University, Changchun, People's Republic of China; 4 College of Animal Science and Technology, Qingdao Agricultural University, Qingdao, People's Republic of China; National Institute on Aging, United States of America

## Abstract

The genomic organization of the IgH (Immunoglobulin heavy chain), Igκ (Immunoglobulin kappa chain), and Igλ (Immunoglobulin lambda chain) loci in the African elephant (*Loxodonta africana*) was annotated using available genome data. The elephant IgH locus on scaffold 57 spans over 2,974 kb, and consists of at least 112 V_H_ gene segments, 87 D_H_ gene segments (the largest number in mammals examined so far), six J_H_ gene segments, a single μ, a δ remnant, and eight γ genes (α and ε genes are missing, most likely due to sequence gaps). The Igκ locus, found on three scaffolds (202, 50 and 86), contains a total of 153 V_κ_ gene segments, three J_κ_ segments, and a single C_κ_ gene. Two different transcriptional orientations were determined for these V_κ_ gene segments. In contrast, the Igλ locus on scaffold 68 includes 15 V_λ_ gene segments, all with the same transcriptional polarity as the downstream J_λ_-C_λ_ cluster. These data suggest that the elephant immunoglobulin gene repertoire is highly diverse and complex. Our results provide insights into the immunoglobulin genes in a placental mammal that is evolutionarily distant from humans, mice, and domestic animals.

## Introduction

The elephant is the biggest terrestrial placental mammal alive today. It belongs to the order Proboscidea and the family elephantidae, which contains only two existing species: the Asian elephant (*Elephas maximus*) and the African elephant (*Loxodonta africana*). The three lineages of this family: *Loxodonta*, *Elephas*, and *Mammuthus* are thought to have originated 4–6 million years ago. Whereas some species of the former two lineages are still alive today, the last representative of the *Mammuthus* lineage, the woolly mammoth (*Mammuthus primigenius*), became extinct very recently (about 3.7 thousand years ago) [1]. Phylogenetic analysis suggest that the elephant is most closely related to living mammals of *Trichechus* (such as the West Indian Manatee, *Trichechus manatus*) and *Procavia* (such as the Rock Hyrax, *Procavia capensis*) [2].

Elephants are reported to be susceptible to a wide variety of infections caused by bacteria [3], [4], viruses [5]–[13], and parasites [14]–[17]. However, there have been very few studies previously performed on the elephant immune system. In addition, little is known about the elephant immunoglobulins, except for serological testing for IgM [18], IgG [19], [20], and IgA [21]. It was reported that there were at least five subclasses of IgG in African elephant sera, with no apparent IgM or IgA [20].

Immunoglobulins are the antigen-recognition molecules of B cells of jawed vertebrates, which usually consist of two identical heavy (H) and two identical light (L) chains. In some exceptional cases, such as shark IgNAR and selected subclasses of camelid IgGs, only heavy chains are used [22]–[24]. Variable regions in the N-terminus of H/L chains are encoded by V_H_/V_L_, D_H_, and J_H_/J_L_ genes to determine the antigen binding site and antibody specificity. However, constant regions in the C-terminus of H/L chains are encoded by IGHC/C_κ_ or C_λ_ genes and are responsible for the immunoglobulin classes and functional activities [25], [26].

In the mammals studied so far, the locus of unique immunoglobulin heavy chain genes and loci of λ and κ light chain genes are commonly organized in a “translocon” pattern [27], [28]. In the heavy chain locus, multiple V_H_, D_H_, and J_H_ gene segments are followed by consecutive μ, δ, γ, ε, and α gene segments [29]. In the λ light chain locus, a cluster of V_λ_ gene segments is followed by multiple sets of clustered J_λ_ gene segments, each linked to a single C_λ_ gene. Differentially, the cluster of V_κ_ gene segments is followed by a cluster of J_κ_ gene segments, and then by a single C_κ_ gene [30].

IgH and IgL loci have been characterized in different mammalian species [31]–[48]. Although the genomic organization of immunoglobulin genes in mammals has remained relatively constant, variation exists in the number of variable, diversity, joining, and constant region genes. Here, we present the genomic organization of the IgH, Igκ, and Igλ loci of the African elephant, annotated on a basis of its genome data.

## Materials and Methods

### The elephant genome sequence

The genome sequence of the African Elephant (*Loxodonta africana*), provided by the Broad Institute via whole genome shotgun, can be obtained from the Ensembl database (http://www.ensembl.org). LoxAfr3, an assembly of the genome of African Elephant, has been sequenced to 7× coverage (loxAfr3, 7× coverage, July 2009). The elephant immunoglobulin gene sequences were retrieved from the UCSC genome browser (http://genome.ucsc.edu/).

### Identification of the elephant Ig genes

Human immunoglobulin gene sequences were used as queries to search the elephant genome scaffolds that contained immunoglobulin genes. A conventional TBLASTN approach was used to identify constant region genes of the elephant immunoglobulins. FUZZNUC, an online software (http://embossgui.sourceforge.net/demo/fuzznuc.html) was used to find adjacent recombination signal sequences (RSSs) for identification of variable, diversity, and joining gene segments. Five or more mismatched bases were allowed to cover all genes. The locations of the annotated elephant gene sequences on the elephant genome are shown in Table S1 (S1-1∼S1-5).

### Sequence alignments

Editing and comparison of sequences were carried out using the DNAstar program. Alignment of multiple sequences was performed using the Clustal W algorithm, then aligned with Clustal X software, and exported by BioEdit software with view conservation by plotting identities to a standard as a dot.

### Dot matrix analysis

A dot matrix analysis (window size 30 bp and mismatch limit 9 bp) was used for comparing two sequences to identify a possible alignment of characters between the sequences.

### Phylogenetic analysis

Phylogenetic studies were carried out using MrBayes3.1 and viewed with the TreeView package. All the trees were obtained with 1 million generations for the chains, a sample frequency of a 100, and a burn in of 2,500 (ngen = 1000000; Samplefreq = 100; burnin = 2,500). The site by site rate variation was set to a gamma distribution (rates = gamma) for all the Bayesian trees and a General Time-Reversible (GTR) (nst = 6) model of substitution was chosen. The sequences from other species used in phylogenetic analyses are presented in Table S2 (S2-1∼S2-2).

### Definition of the V_H_/V_L_ gene families

In mammals, germline V_H_ and V_L_ gene segments can be grouped into families based on their nucleotide sequence similarity [49]. The established criteria are that the same family members share more than 80% nucleotide similarity, those with less than 70% similarity are put into different families, and those possessing between 70% and 80% similarity are inspected on a case-by-case basis [50]. In our analysis, we placed V_H_ and V_L_ segments having similarity greater than 70% into the same family.

## Results

### Elephant immunoglobulin heavy chain genes

#### IgH locus

**Figure 1:**
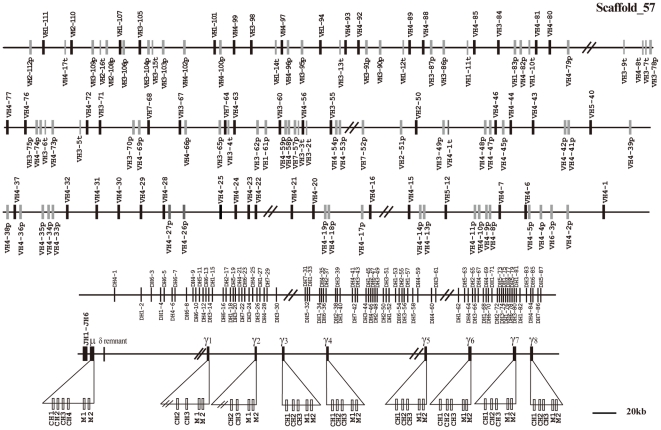
The elephant IgH locus. The elephant IgH locus is localized to scaffold 57. The length is approximately 2,974 kb from the most 5′ V_H_ segment (V_H_2-112p) to the most 3′γ segment. Filled bars: potentially functional V_H_ genes; open bars: V_H_ pseudogenes: open bars and V-p; thin bars: truncated V_H_ segments and V-t; D_H_: diversity genes; J_H_: joining genes; μ: IgM coding gene; δ: IgD remnant; and γ: IgG coding gene. V_H_ and D_H_ genes are numbered based on the families to which they belong and their positions in the locus. The number before the dash (V_H_n or D_H_n) indicates the family, while the number after dash represents the genomic position. J_H_ genes are numbered based on the order of their locations in the locus. Double slashes indicate gaps >10 kb. Alpha and epsilon genes are not displayed on the map, but this does not imply their absence in the elephant IgH locus.

The public elephant genome assembly used in this study was loxAfr3, which is an assembly of the genome of the African Elephant (*Loxodonta africana*), sequenced to 7× coverage. The high genome coverage of this assembly confers a high reliability on the gene analysis. BLAST searching localized the elephant IgH locus to genomic scaffold 57. It spans approximately 2,974 kb from the most 5′ V_H_ segment (V_H_2-112p) to the most 3′ γ gene (Fig. 1). A single μ and eight γ genes were identified in this scaffold. Neither ε nor α genes could be found, most likely due to sequence gaps.

#### Constant region genes

Like other mammalian species, the elephant μ gene contains four CH and two transmembrane exons. A sequence comparison of μ genes among thirteen vertebrate species demonstrated that the critical amino acids for immunoglobulin folding, Cysteine (C) and Tryptophan (W) [51], were highly conserved in elephants (Figure S1). In addition, the elephant IgM constant region showed the highest amino acid sequence identity to human (63.8%), and the least to echidna (50.8%).

Most mammals also express a δ gene, which is always situated immediately downstream of the μ, and the distance between μ and δ usually does not exceed 7 kb. A BLAST search against the elephant whole genome using both DNA and amino acid sequences of the δ genes of other mammalian species showed no intact δ gene. However, approximately 10 kb downstream of the elephant µ (no sequence gaps for 90 kb downstream), we identified a short fragment encoding a polypeptide (Figure S2) homologous to the IgD CH3 domain of other mammals. This was done by a thorough examination of amino acid sequences encoded by the DNA sequences between μ and γ1 (based on translation of all reading frames of both sense and anti-sense sequences). An alignment of the elephant IgD remnant and the IgD CH3 domains of several mammalian species is presented in Figure S2. This indicates that the gene has been highly mutated and pseudogenized in the elephant.

**Figure 2:**
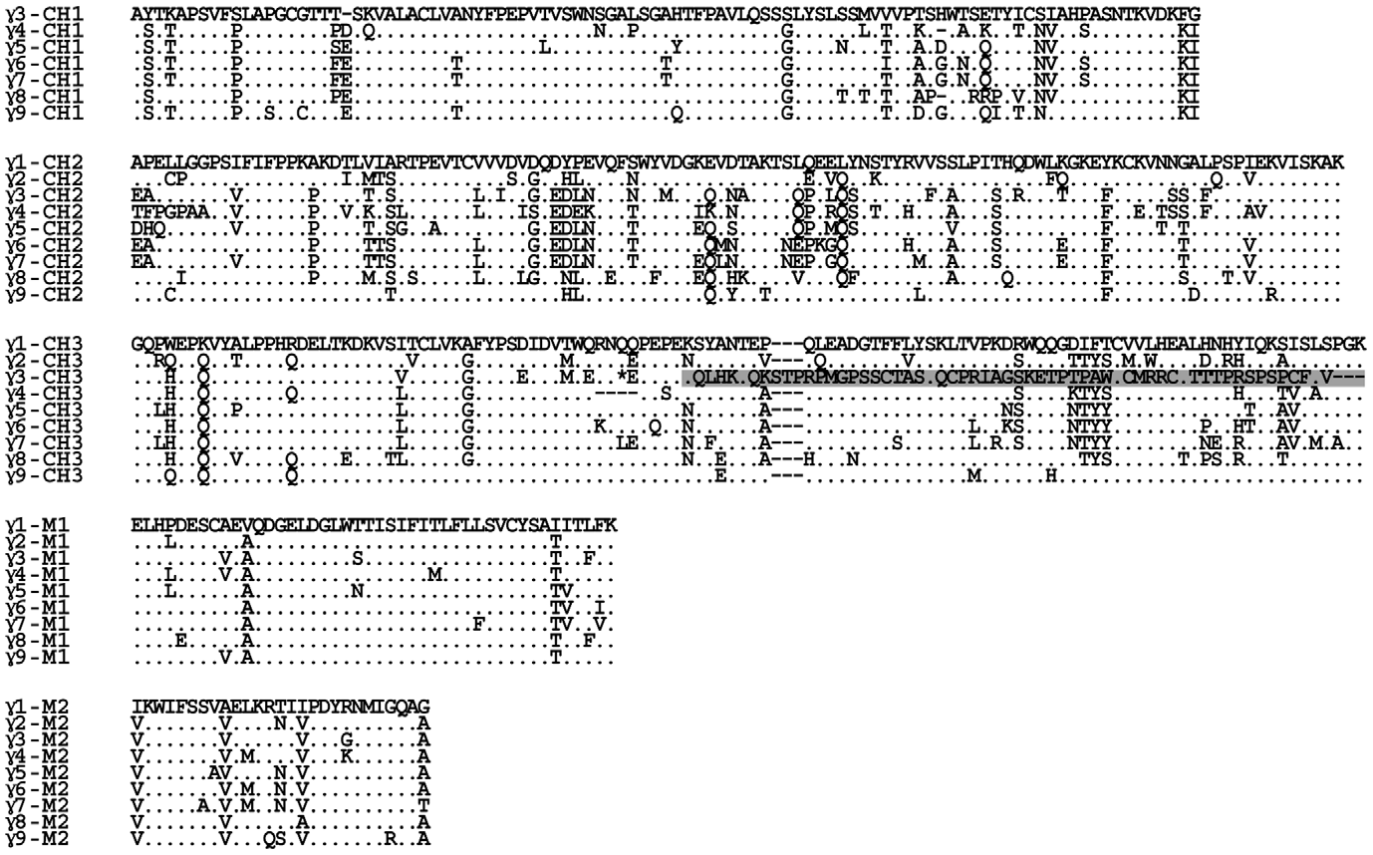
Alignment of the amino acid sequences of the nine elephant γ genes. The deduced amino acid sequences of three γ chain-coding exons and two membrane exons of nine elephant γ genes are aligned. The first exons (CH1) of γ1 and γ2 are both missing due to gaps. A premature stop codon is marked with a star and a frameshift mutation is marked by shadowing in the CH3 exon of γ3. In each panel, the amino acid residues that are identical to the top sequences are represented by dots. The dashes were used to adjust the alignment. The hinge exons are not included in the figure.

In addition to the eight γ genes (γ1 to γ8) in scaffold 57 (Fig. 1), an additional γ gene (tentatively named as γ9) was identified in scaffold 495 (data not shown), which spans 77 kb. Scaffold 495 is not assembled together with scaffold 57; therefore, γ9 could potentially be either an additional subclass encoding gene or an allelic variant. The identification of multiple IgG subclass-encoding genes is in accordance with a previous report, which indicated that there were at least five subclasses of IgG in African elephant sera [20]. Sequence analysis showed no additional Ig genes in genomic scaffold 495, except for the γ9 gene. The greatest variation among mammalian IgG subclasses is usually concentrated in their hinge regions [52]–[54]. However, no elephant IgG cDNA sequences have been sequenced, it is very difficult to accurately assess the hinge regions of the elephant IgG heavy chains. The hinge region is usually encoded on a separate exon that could not be identified in the elephant due to the low level of conservation and the absence of cDNA sequences. An amino acid alignment of the nine elephant IgG subclasses is presented in Fig. 2. The first exons (CH1) of γ1 and γ2 are both missing because of gaps. The CH3 exon of γ3 is pseudogenized because of a premature stop codon (marked with a star in Fig. 2), and a frame-shift mutation (marked with shadowing in Fig. 2) caused by nucleotide (adenine) insertions at positions 148 and 158, respectively. To clarify the relationship among γ chains from mammalian species, a phylogenetic tree of IgG CH2 and CH3 exons was constructed and is shown in Figure S3. The elephant γ genes form a distinct cluster. This is consistent with previous analysis, which showed that the divergence of IgG subclasses occurred after speciation [52].

**Figure 3:**
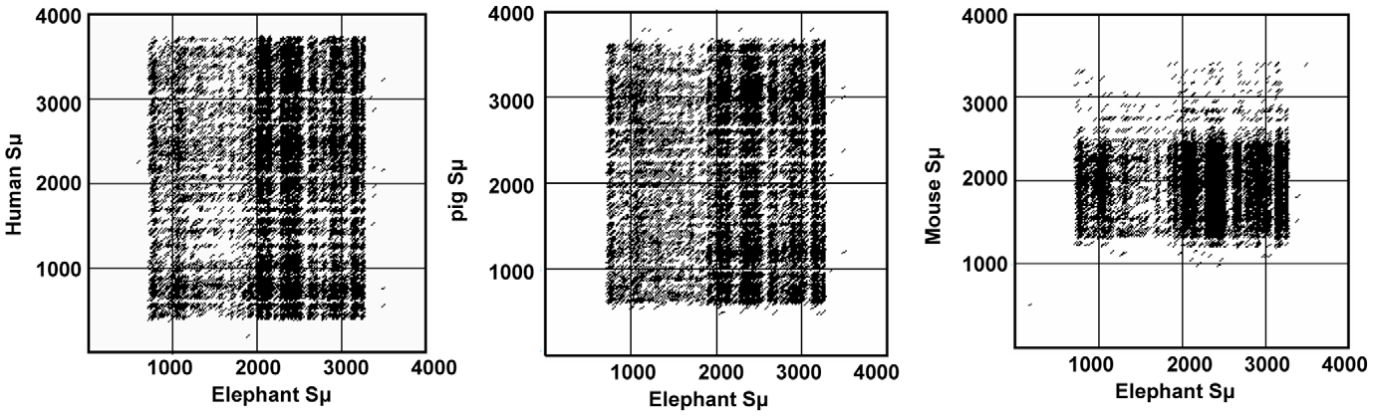
Dot plot comparison of the elephant S_μ_ with human, pig, and mouse S_μ_ regions. The dots represent homologies with a search length of 30 bp and maximum of 9 bp mismatches. The elephant **S_μ_** region shows substantial nucleotide sequence homology with those of human, mouse, and pig.

**Figure 4:**
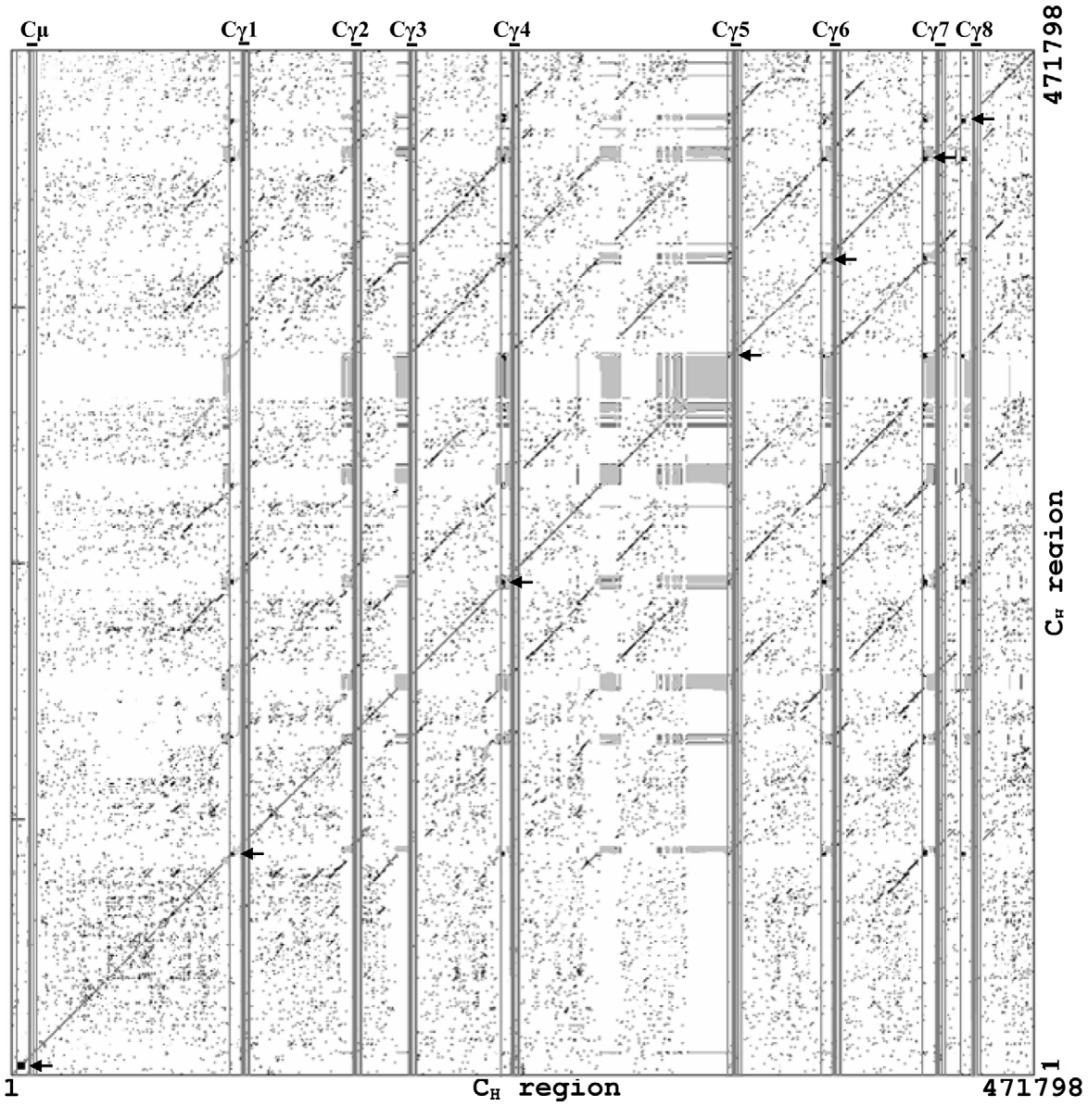
Dot plot comparison of the elephant C_H_ (μ and γ) region. A dot matrix representing repetitive sequences of elephant C_H_ (μ and γ) genes. Switch regions are indicated by black-squared boxes and marked with arrowheads, and gaps are indicated by grey-squared boxes. Positions of C_H_ genes are indicated as vertical lines. The dots represent homologies with a search length of 30 bp and maximum of 9 bp mismatches.

Dot matrix analysis of the elephant IgH locus showed there are switch regions upstream of the μ gene and six γ genes (γ1, γ4, γ5, γ6, γ7, and γ8), as in humans and mice [55], [56]. The switch regions of γ2, γ3, and γ9 could not be identified, most likely due to sequence gaps. Structurally, the switch regions, as in other species, are all composed of pentameric repeats (GGGCT and GAGCT). The elephant S_μ_ region shows substantial nucleotide similarity with those of human, mouse, and pig (Fig. 3). The six elephant S_γ_ regions are similar, but share little sequence similarity with human and mouse S_γ_ (Fig. 4 and data not shown).

#### V_H_ gene segments

**Figure 5:**
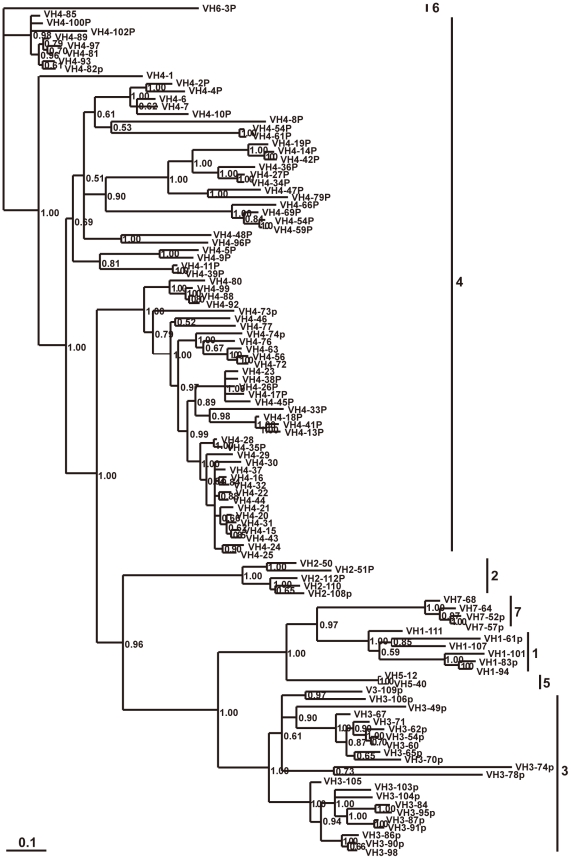
Phylogenetic analysis of the 112 elephant V_H_ genes. A phylogenetic tree of nucleotide sequences of 112 elephant V_H_ segments was constructed. The seven identified V_H_ gene families are labeled with Arabic numerals. The credibility value for each node is shown.

**Figure 6:**
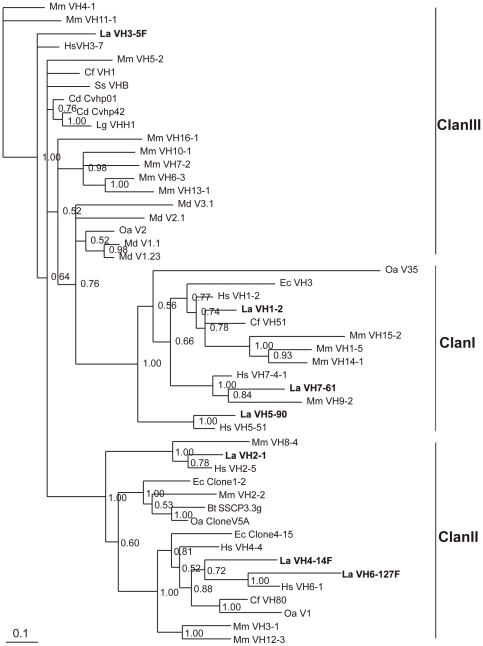
Phylogenetic analysis of mammalian V_H_ genes. Representatives of the seven elephant V_H_ families are clustered with their human counterparts. The three mammalian V_H_ clans are labeled with Roman numerals. The credibility value for each node is shown.

A total of 112 V_H_ segments were identified in the elephant IgH locus. 51 of them appear to be potentially functional, because they have leader exons, normal open reading frames (ORF), downstream RSSs, and V gene domain (framework regions (FRs) and complementarity determining regions (CDRs)). The remaining 61 segments contain either in-frame stop codons or frameshifts, and are thus designated as pseudogenes. In addition, there are 17 partial segments of about 200 bp in length, which are regarded as truncated V_H_ sequences. There are gaps above 10 kb in the elephant genome among the V_H_ gene segments (Fig. 1), suggesting that there might be more V_H_ segments. To examine the relationships among the elephant germline V_H_ segments, pseudogenes as well as functional genes were used to construct a phylogenetic tree (Fig. 5). The seven identified V_H_ gene families (1, 2, 3, 4, 5, 6, and 7) were confirmed to be homologous with the corresponding human V_H_ gene families. The elephant V_H_4 family contains the most members (72 V_H_ segments), which could be further divided into three groups (Fig. 5). We chose representative V_H_ sequences from elephant and other mammals, covering almost all V_H_ families identified, to construct phylogenetic trees (Fig. 6). The elephant VH genes clearly fall into the three previously known VH clans.

#### D_H_ gene segments

**Figure 7:**
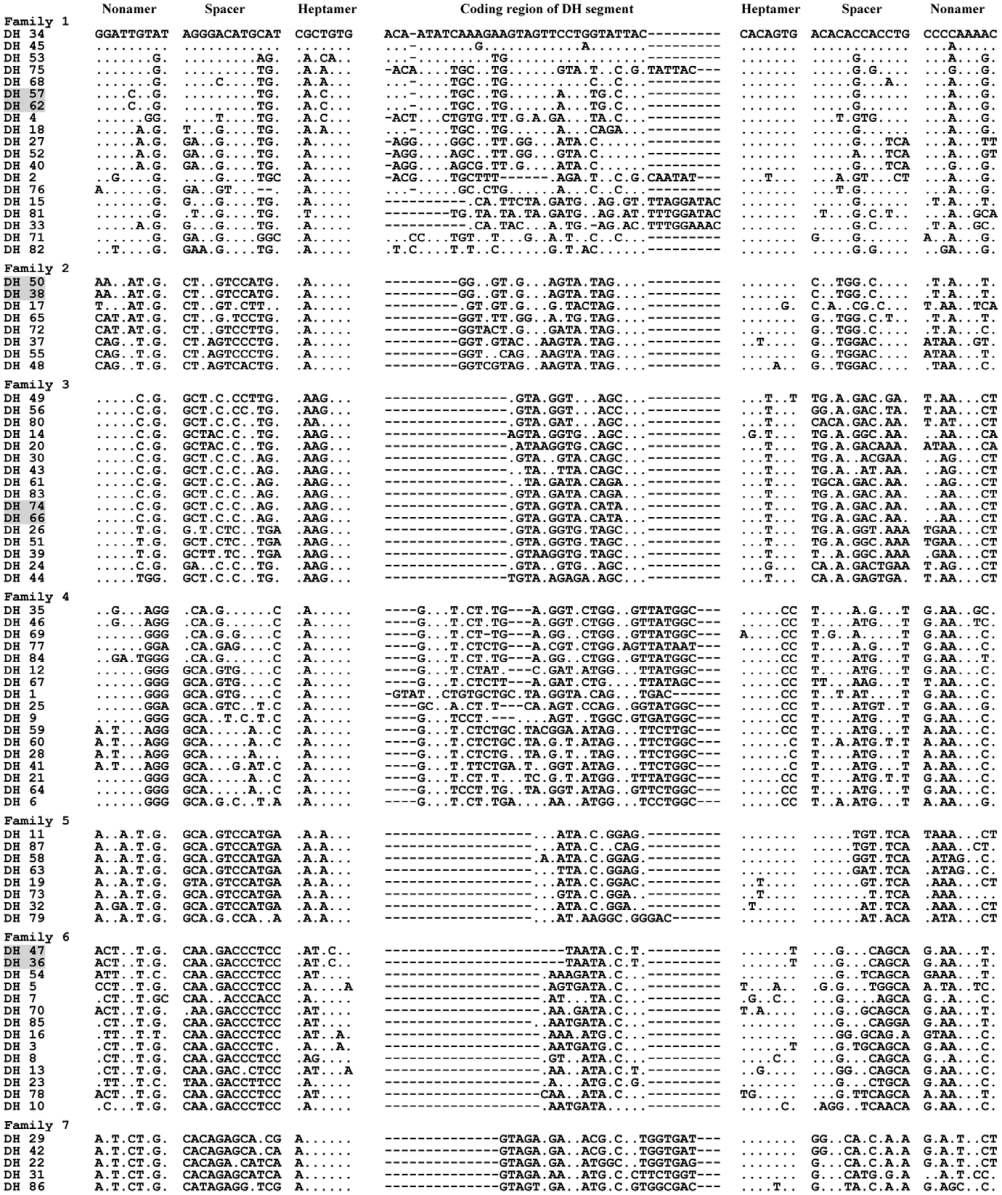
Alignment of nucleotide sequences of seven elephant germline D_H_ families. Seven families representing elephant 87 germline D_H_ segments are aligned. Nucleotides that are the same as the top segment, D_H_34, are indicated with dots. Dashes mean gaps introduced to make the alignment. D_H_57 and 62, D_H_50 and 38, D_H_74 and 66, and D_H_47 and 36 are shadowed as they share identical sequences. Coding regions of DH segments are separated from recombination signal sequences (RSSs) (nonamer, spacer, and heptamer).

In the elephant IgH locus, 87 D_H_ segments were identified and are presented in Figure S4 (S4-1∼S4-10). It should be noted that there might be more D_H_ segments because of the existence of sequence gaps. Except for DH76, which has a 10 bp spacer, all the D_H_ segments are flanked by characteristic heptamers and nonamers separated by 12-bp spacers. The potential coding regions of D_H_ segments are 10–37 bp in length (Figure S4, S4-1∼S4-10). It has been suggested that coding regions of D_H_ segments of humans can be described by the characteristics of their amino acids [57]. Inspection showed that a great number of polar/hydrophobic amino acids or stop codons occur widely in elephant D_H_ coding regions (data not shown). In humans and mice, the germline D_H_ segments can be classified into families based on the extent of sequence similarity [58], [59]. Analysis of nucleotide similarity in the coding regions and flanking RSSs indicated that the 87 elephant D_H_ segments could be divided into seven families. Members within the same family share at least 70% nucleotide identity (data not shown), while some members in a family have completely identical sequences (these are shadowed in Fig. 7). We present the sequence alignment of the seven families in Fig. 7, which shows that each family contains characteristic sequence intervals that are distinct from other families.

#### J_H_ gene segments

**Figure 8:**
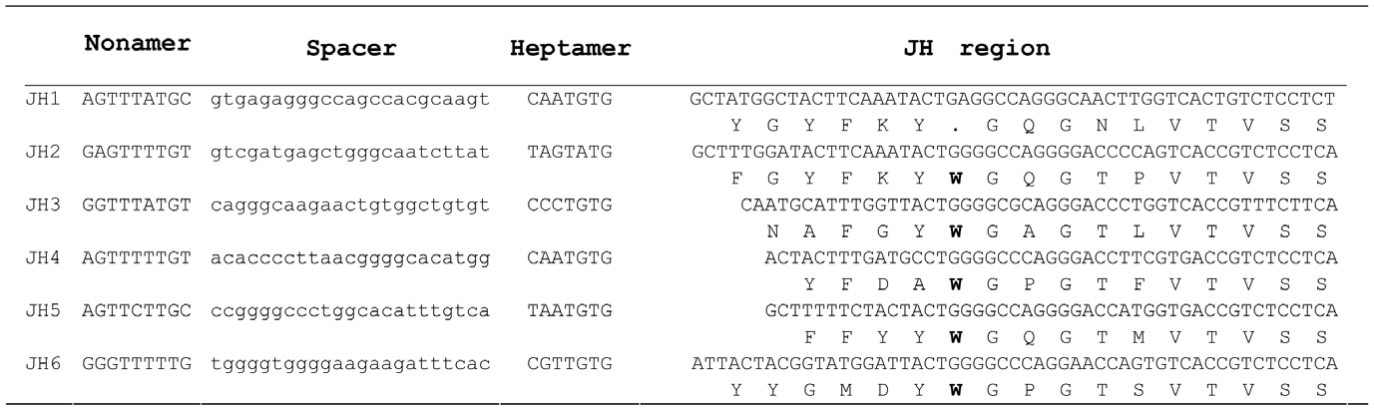
The six elephant germline J_H_ gene segments. Nucleotide and deduced amino acid sequences of six J_H_ segments, along with RSSs, are shown. The amino acid residue W is replaced by a stop codon in the J_H_1 segment.

There were six germline JH gene segments found in the elephant IgH locus (Fig. 8). All the JH segments had conserved nucleotide sequences at the 3′ end. JH1 was pseudogenized by replacement of a Tryptophan (W) residue by a stop codon.

### Elephant immunoglobulin light chains

#### κ chain

**Figure 9:**
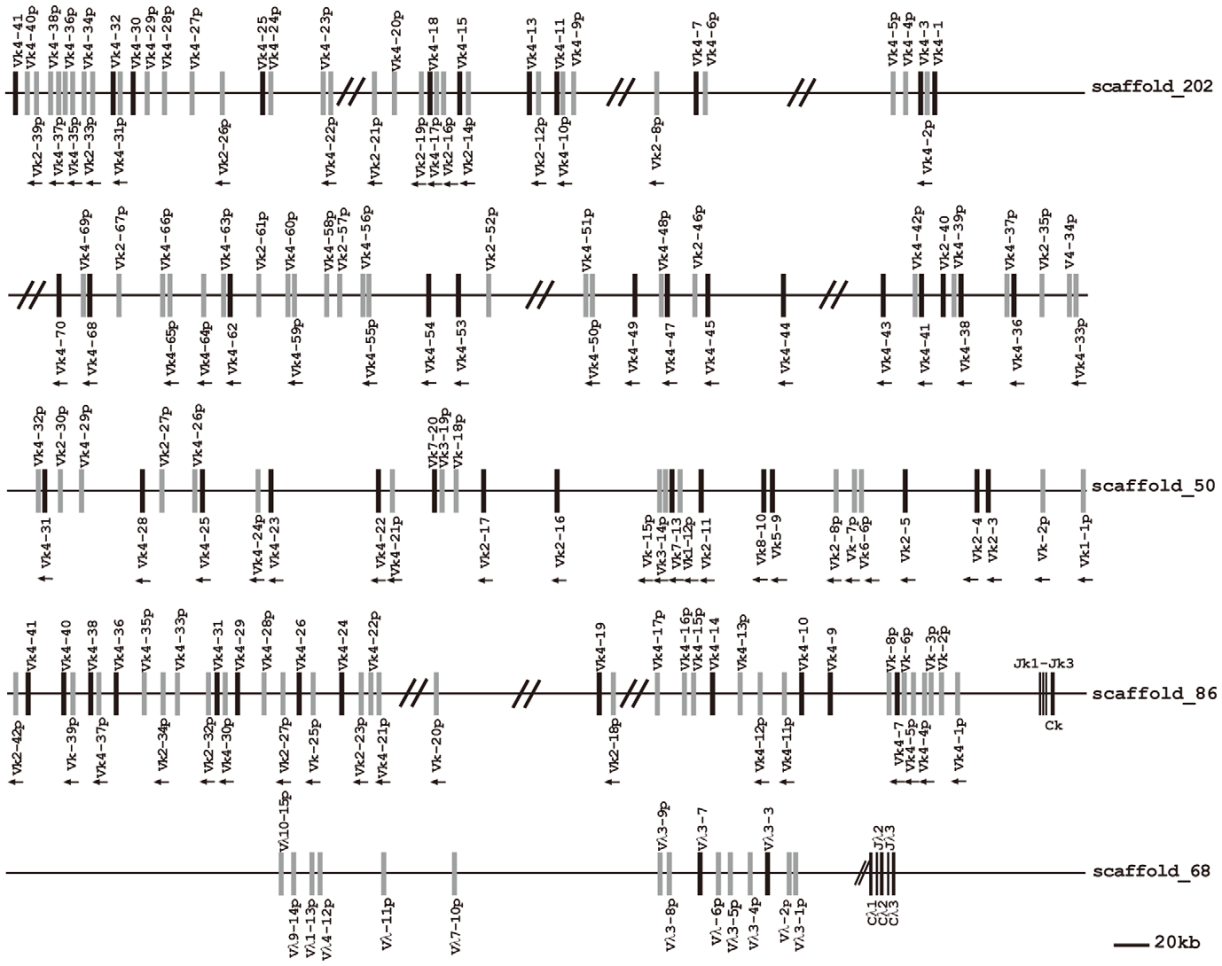
The elephant IgL locus. The elephant Igκ locus is distributed over three scaffolds (202, 50, and 86), and the Igλ locus is located on scaffold 68. Overall configurations are drawn approximately to scale. The potentially functional V_κ_ and V_λ_ genes are shown as filled bars, while pseudogenes are represented by open bars and indicated with the letter p. Double slashes indicate gaps >10 kb. The unidirectional arrowheads below V_κ_ gene segments on scaffold 86 indicate that their transcriptional direction is opposite to downstream J_κ_ segments. However, the unidirectional arrowheads on scaffolds 202 and 50 do not represent different transcriptional directions from the identified J_κ_ gene segment; they merely indicate a transcriptional direction different from that of the remaining V_κ_ gene segments in the scaffold.

**Figure 10:**
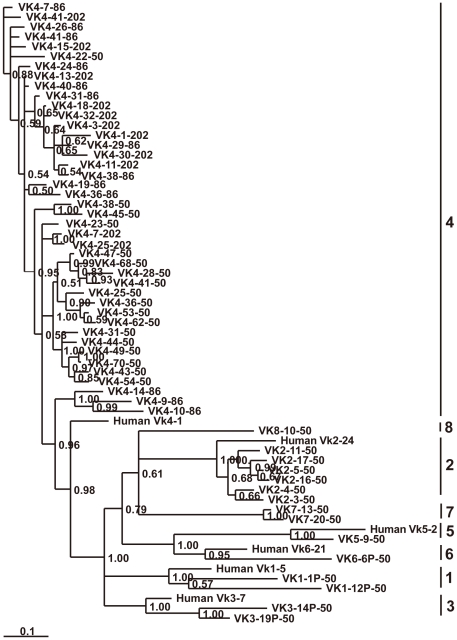
Phylogenetic analysis of the 58 elephant V_κ_ genes. A phylogenetic tree of the nucleotide sequences of 58 elephant V_κ_ segments was constructed. The eight V_κ_ gene families are labeled with Arabic numerals. The credibility value for each node is shown.

Immunoglobulin κ chain genes of elephant were identified on three scaffolds: 202, 50, and 86. A schematic diagram is shown in Fig. 9. Of the 153 germline V_κ_ segments from the three scaffolds, 53 were regarded as potentially functional genes and 100 as pseudogenes. Based on sequence similarity analysis, 142 of the V_κ_ segments can be assigned to eight families (V_κ_1∼V_κ_8) (Table S3), which contain 2, 31, 2, 102, 1, 1, 2, and 1 members, respectively. The remaining 11 V_κ_ pseudogenes could not be assigned to any family because they share less than 70% nucleotide similarity with any other V_κ_ gene segment. A phylogenetic tree of the elephant V_κ_ functional genes is shown in Fig.10. The six elephant V_κ_ families (V_κ_1∼V_κ_6) correspond to the six human V_κ_ gene families. In addition, scaffold 86 includes 24 V_κ_ segments showing the same transcriptional orientation as the J_κ_ and C_κ_, and 18 V_κ_ segments showing a reverse transcriptional direction. Three J_κ_ segments and one C_κ_ gene on scaffold 86 are displayed in Figure S5. In addition, V_κ_ segments located on scaffolds 202 and 50 also possess two different transcriptional directions.

#### λ chain

**Figure 11:**
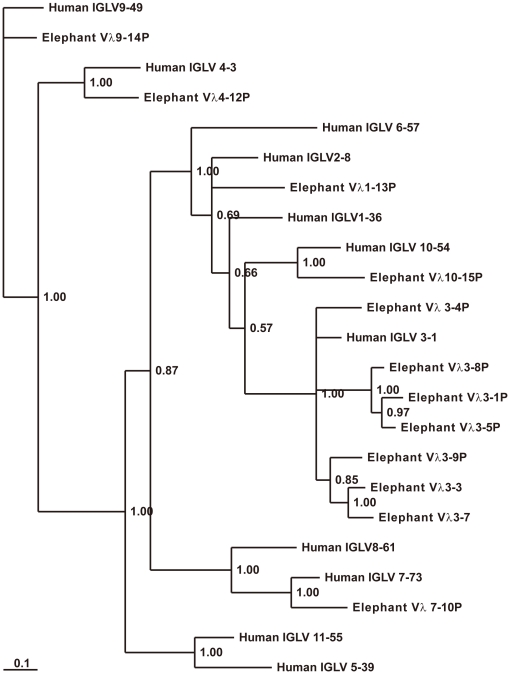
Phylogenetic analysis of the 12 elephant V_λ_ genes. A phylogenetic tree of the nucleotide sequences of the 12 elephant V_λ_ segments was constructed. The 12 elephant V_λ_ gene segments belong to six families, which are clustered with the human V_λ_ 1, 3, 4, 7, 9, and 10 families, respectively. The credibility value for each node is shown.

Scaffold 68 was determined to contain the elephant λ light gene complex (Fig. 9). Sequences analysis revealed that the 12 elephant V_λ_ gene segments belonged to six families (Fig. 11), which were homologous with the human V_λ_ 1, 3, 4, 7, 9 and 10 families. The remaining three V_λ_ pseudogenes could not be assigned to any family because they share less than 70% nucleotide similarity with any other V_λ_ gene segment. The three elephant V_λ_ families consists of seven members. In contrast to V_κ_, all the V_λ_ segments possess an identical transcriptional polarity to the downstream J_λ_ segments. In addition, only V_λ_3-3 and V_λ_3-7 are identified as potentially functional genes. At the 3′ end of the locus, three constant region genes are organized in tandem, where both C_λ_2 and C_λ_3 are preceded by a J_λ_. The J_λ_ segment before C_λ_1 is missing because of a sequence gap. Three C_λ_ genes show approximately 90% amino acid identity. The sequences of two J_λ_ segments and three C_λ_ genes are presented in Figure S5.

## Discussion

In this study, we have made a preliminary analysis of the immunoglobulin genes in the elephant using the recently released elephant genome, revealing that the elephant IgH locus conforms to the “translocon” pattern. Compared with human IgH locus, which occupies a 1.25 Mb region [60], elephant IgH locus appears to span larger genomic region (approximately 3 Mb).

We translated the nucleotide sequences between the μ and γ1 genes in all three reading frames in both the positive and negative directions. By blasting the nucleotide and corresponding amino acid sequences against the NCBI database, only the IgD-CH3 remnant was identified.

With the exception of marsupials [61], [62], most placental and even monotreme mammals studied so far have been shown to have multiple IgG subclasses encoded by independent sets of exons [63]. The elephant genome contains nine IgG genes, although it is not known whether all of them are functional. This number is larger than that in any placental mammals so far examined (ranging from 1 to 7) [43], [64]–[69], providing another remarkable example for IgH chain constant region diversity in mammals.

Our analysis also suggested a high degree of complexity in the elephant IgVH locus. At least 112 V_H_ segments constitute the elephant germ-line V_H_ repertoire. According to the number of V_H_ gene families, placentals studied so far could be divided into two groups. The multiple gene families group includes mice (16 families), human (seven families), and horse (seven families). The few gene families or single gene family group includes dog (three families), rabbits (one family), cattle (one family), camel (one family), and swine (one family) [70]–[78]. The elephant, having 7 V_H_ gene families, should be put into the first group. The mammalian V_H_ families can be further classified into three clans: I, II, and III, which have co-existed in the genome for more than 400 Myr [79]. Similar to those of humans, the elephant V_H_ families also conform to three clans: families 1, 5, and 7 form clan I, families 2, 4, and 6 form clan II, and family 3 forms clan III. The largest group of elephant V_H_ genes is the V_H_4 family of clan II. It has been demonstrated that the unique V_H_ family identified in cattle belonged to clan II [75], [77]. In sheep, most V_H_ genes are also categorized into clan II [80]. Based on a recent report, clan II also appeared to be the largest group in the horse [41], indicating that the herbivore animals may prefer to use the clan II V_H_ genes.

Close attention should also be paid to the elephant D_H_ locus, where at least 87 germline D segments could be mapped to a 450-kb DNA region; the largest number in mammals examined so far. The presence of more D_H_ segments may greatly increase the Ig diversity generated through DNA rearrangement. The size of the elephant D_H_ coding regions ranges from 10 to 37 bp, similar to that of human (11 to 37 bp) [57]. Further inspection revealed that the elephant D_H_ segments were translated in three reading frames abundant in polar/hydrophobic amino acids, which is different to dog [78], horse [41], mouse [81], rabbit [82], and chicken [83], which show preferences for neutral (polar/hydrophilic) amino acids.

For the light chain genes, elephant V_κ_ germline genes are more abundant than V_λ_ (53 functional V_κ_ genes *vs.* 2 functional V_λ_ genes). Different mammalian species possess different ratios of V_κ_ and V_λ_. In humans, roughly 60% of the variable light chain repertoire is κ (40 functional V_κ_ genes *vs*. 30 functional V_λ_ genes). The germline V_κ_ genes of mice are dominant by as much as 95% or more [84]. It has been proposed that the preferential use of light chain isotypes at the protein level may be correlated with the overall number of V gene segments [84]. It is thus possible that the κ chain predominates over the λ chain at the protein level in elephants.

Interestingly, a great number of pseudogenes exist in the elephant V_H_ (61/112), V_κ_ (100/153), and V_λ_ (13/15) loci. In some species, the base-pair changes could be inferred using an existing pseudogene or germline gene as a template, and therefore pseudogenes in the V loci constitute a potential donor pool for gene conversion to generate immunoglobulin diversity [85]–[88]. A great number of V pseudogenes may contribute to the immunoglobulin diversity in elephants.

The study of structure and organization of the immunoglobulin gene loci is vital to the understanding of the nature of antibody molecules. This study provides information for comparative studies of mammalian Ig genes, as well as data for further studies of the elephant immunoglobulin genes.

## Supporting Information

Figure S1 **Alignment of IgM amino acid sequences from several vertebrate species.** Elephant IgM was compared with a panel of vertebrate IgM sequences. Dots indicate similar residues as in elephant μ, whereas dashes indicate gaps introduced for optimal alignment. The cysteine residues C and W important for intra-domain disulfide bonds are shown on the first line of the alignment.
(TIF)

Figure S2 **Alignment of the elephant IgD remnant with the IgD CH3 domains of several mammalian species.** Amino acid residues that are identical to the top counterpart in every panel are shown as dots; Gaps and missing data are indicated by hyphens. Stop codons are indicated by stars.
(TIF)

Figure S3 **Phylogenetic tree of the immunoglobulin gamma heavy chains of some mammalian species.** The phylogenetic tree was constructed from the amino acid sequences of the CH3 exons of the immunoglobulin gamma heavy chains of various mammalian species. The credibility value for each node is shown.
(TIF)

Figure S4 **87 Elephant germline D_H_ segments.** The nonamers (9-mer) and heptamers (7-mer) are displayed. Heptamer components that are different from the consensus (5′: CACTGTG and 3′: CACAGTG) are shadowed. The deduced amino acids of all three reading frames of the coding region of D segments are shown. Except D_H_, which has a 10 bp spacer, all the D_H_ segments were attached by 12 bp spacer.
(TIF)

Figure S5 **The alignment of amino acid sequences of J and C genes from elephant IgL chains.** A, alignment of the deduced amino acid sequences of the three elephant J_κ_ gene segments. B, alignment of the amino acid sequences of the C_κ_ proteins from several mammalian species. C, alignment of the deduced amino acid sequences of the two elephant J_λ_ gene segments. D, alignment of the deduced amino acid sequences of three elephant C_λ_ genes and several mammalian species C_λ_ genes. Amino acid residues that are identical to the top counterpart in every panel are shown as dots; Gaps and missing data are indicated by hyphens.
(TIF)

Table S1 **The elephant immunoglobulin heavy chain and light chain DNA segments located in scaffolds 57, 202, 50, 86, and 68.**
(RAR)

Table S2 **GenBank accession numbers or references of the gene sequences from other species used in this paper.**
(RAR)

Table S3 **The eight elephant V_κ_ gene families from scaffolds 202, 50, and 86.**
(TIF)
