# Supplementary figures and images for: A Preliminary Analysis of the Immunoglobulin Genes in the African Elephant (Loxodonta africana)
Source: PLoS One. 2011 Feb 25;6(2):e16889. doi: 10.1371/journal.pone.0016889 (PMC3045440; doi:10.1371/journal.pone.0016889)

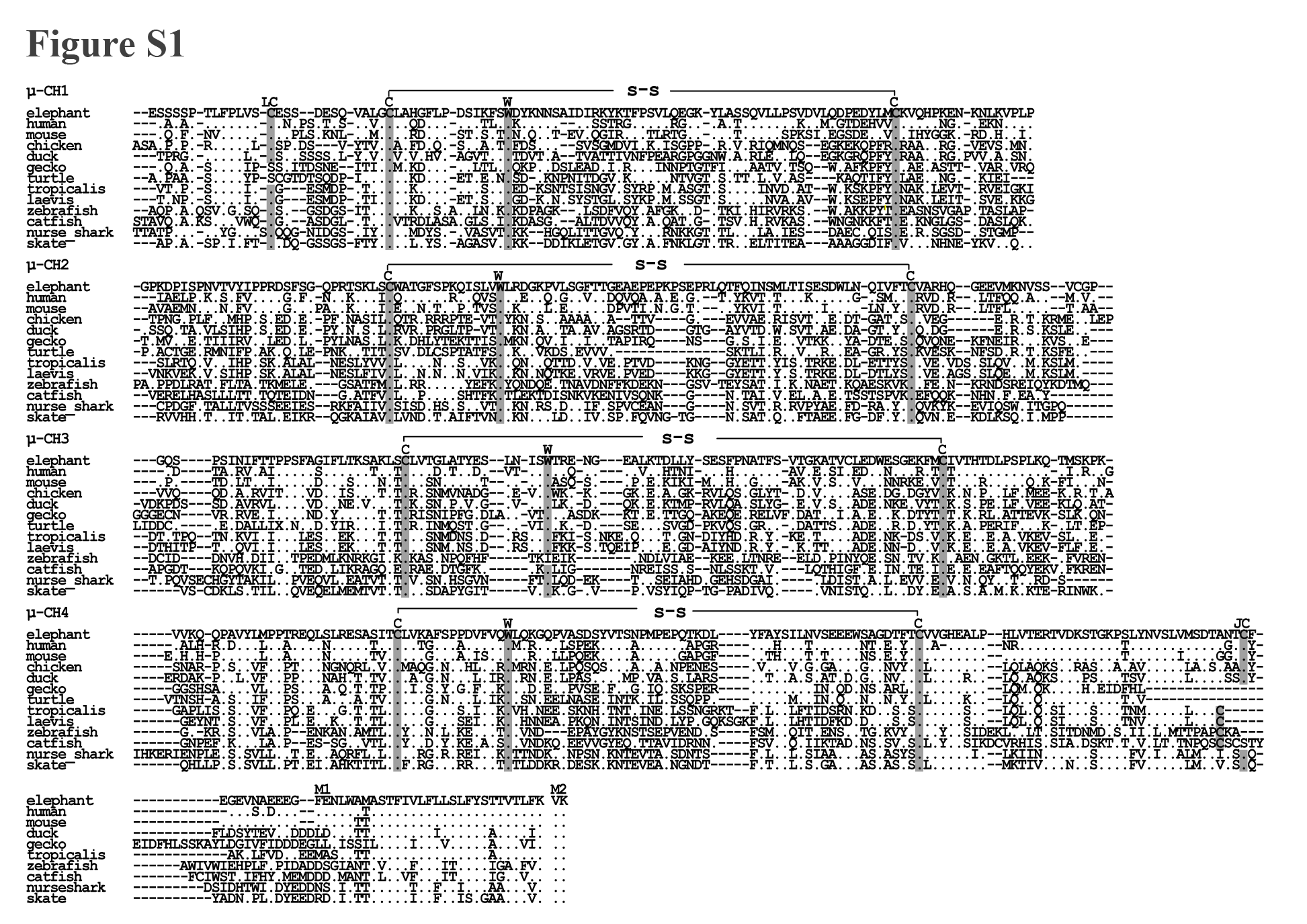

Supplement: Figure S1 — Alignment of IgM amino acid sequences from several vertebrate species. Elephant IgM was compared with a panel of vertebrate IgM sequences. Dots indicate similar residues as in elephant μ, whereas dashes indicate gaps introduced for optimal alignment. The cysteine residues C and W important for intra-domain disulfide bonds are shown on the first line of the alignment. (TIF) [file pone.0016889.s001.tif]

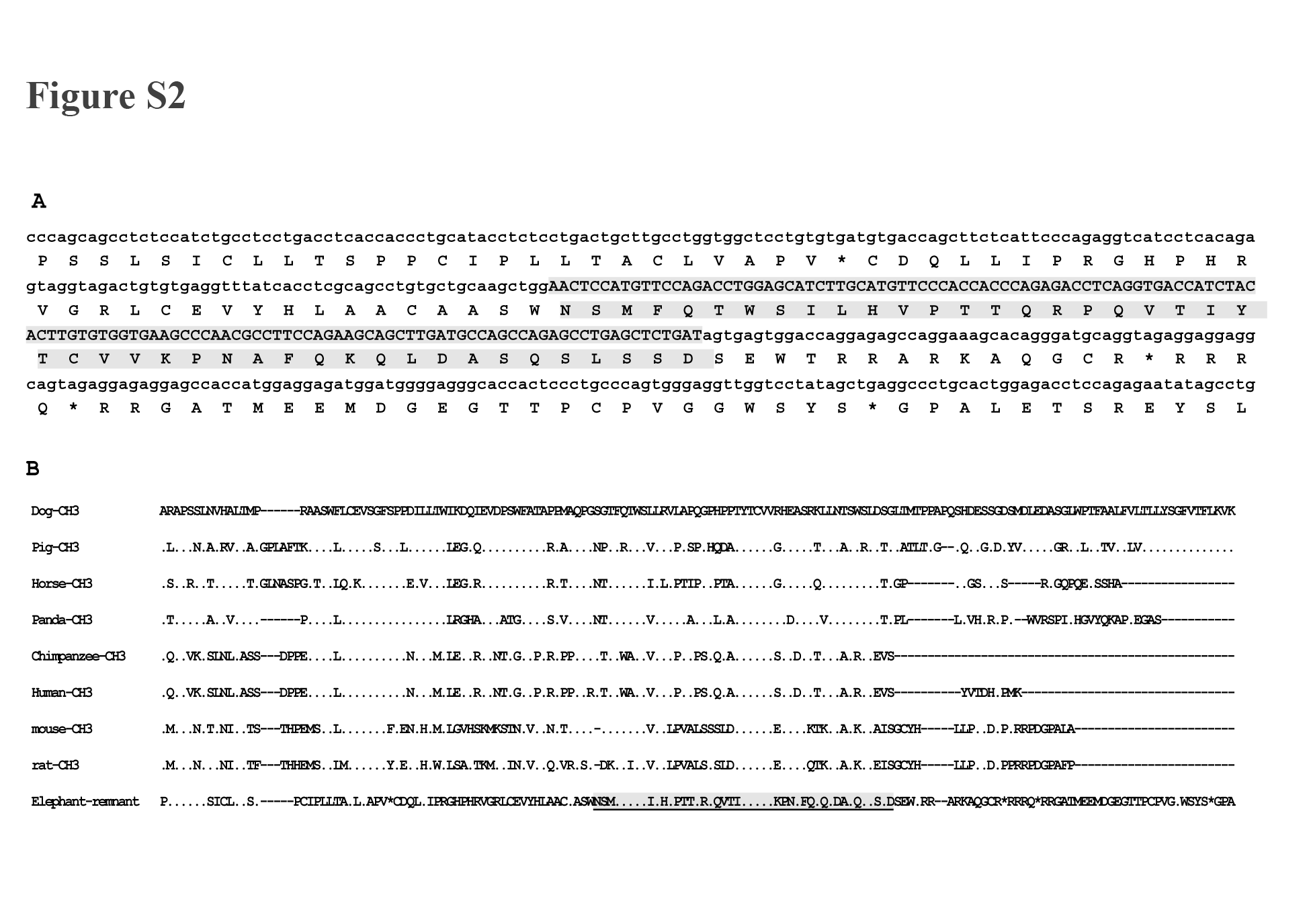

Supplement: Figure S2 — Alignment of the elephant IgD remnant with the IgD CH3 domains of several mammalian species. Amino acid residues that are identical to the top counterpart in every panel are shown as dots; Gaps and missing data are indicated by hyphens. Stop codons are indicated by stars. (TIF) [file pone.0016889.s002.tif]

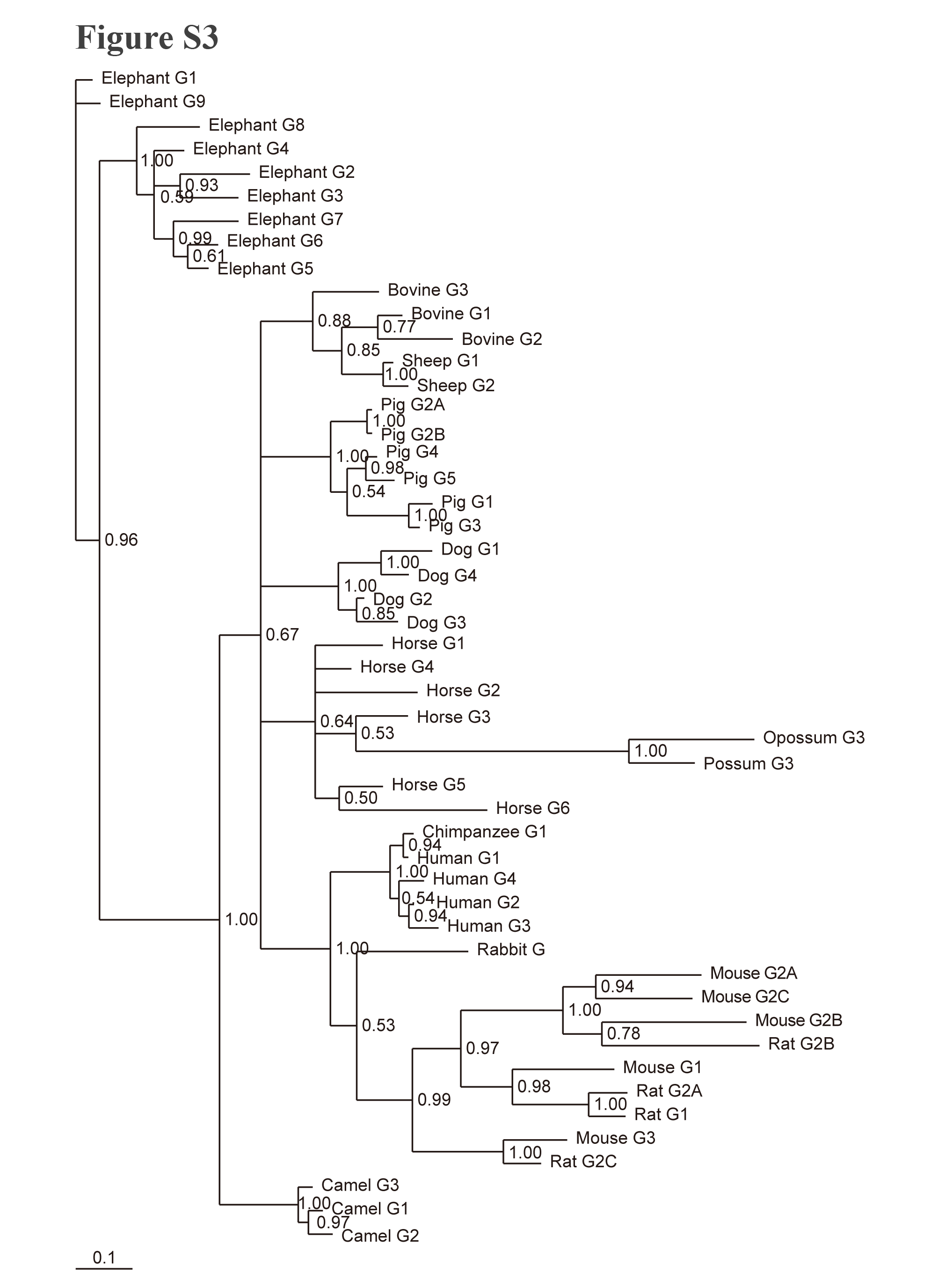

Supplement: Figure S3 — Phylogenetic tree of the immunoglobulin gamma heavy chains of some mammalian species. The phylogenetic tree was constructed from the amino acid sequences of the CH3 exons of the immunoglobulin gamma heavy chains of various mammalian species. The credibility value for each node is shown. (TIF) [file pone.0016889.s003.tif]

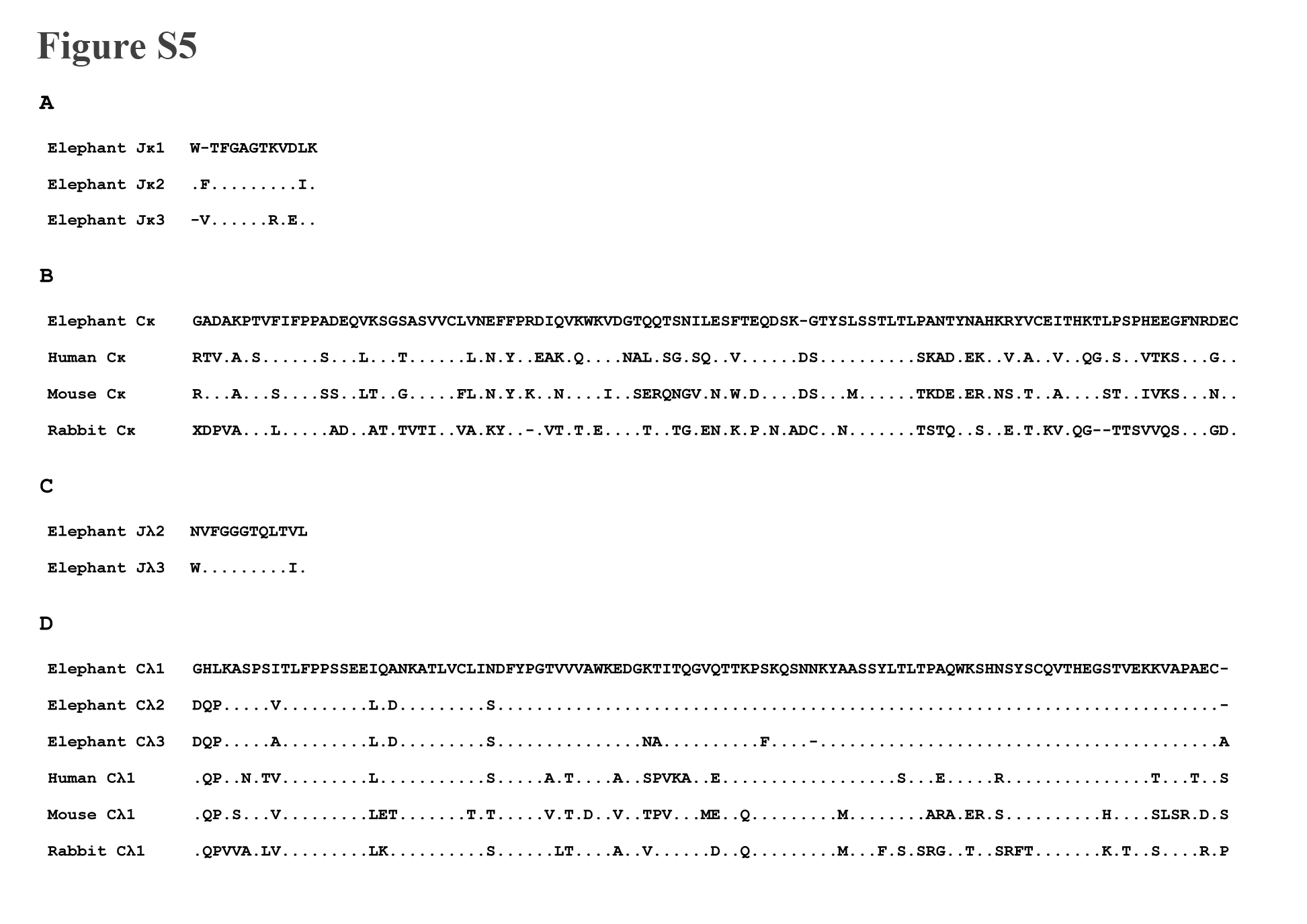

Supplement: Figure S5 — The alignment of amino acid sequences of J and C genes from elephant IgL chains. A, alignment of the deduced amino acid sequences of the three elephant Jκ gene segments. B, alignment of the amino acid sequences of the Cκ proteins from several mammalian species. C, alignment of the deduced amino acid sequences of the two elephant Jλ gene segments. D, alignment of the deduced amino acid sequences of three elephant Cλ genes and several mammalian species Cλ genes. Amino acid residues that are identical to the top counterpart in every panel are shown as dots; Gaps and missing data are indicated by hyphens. (TIF) [file pone.0016889.s005.tif]

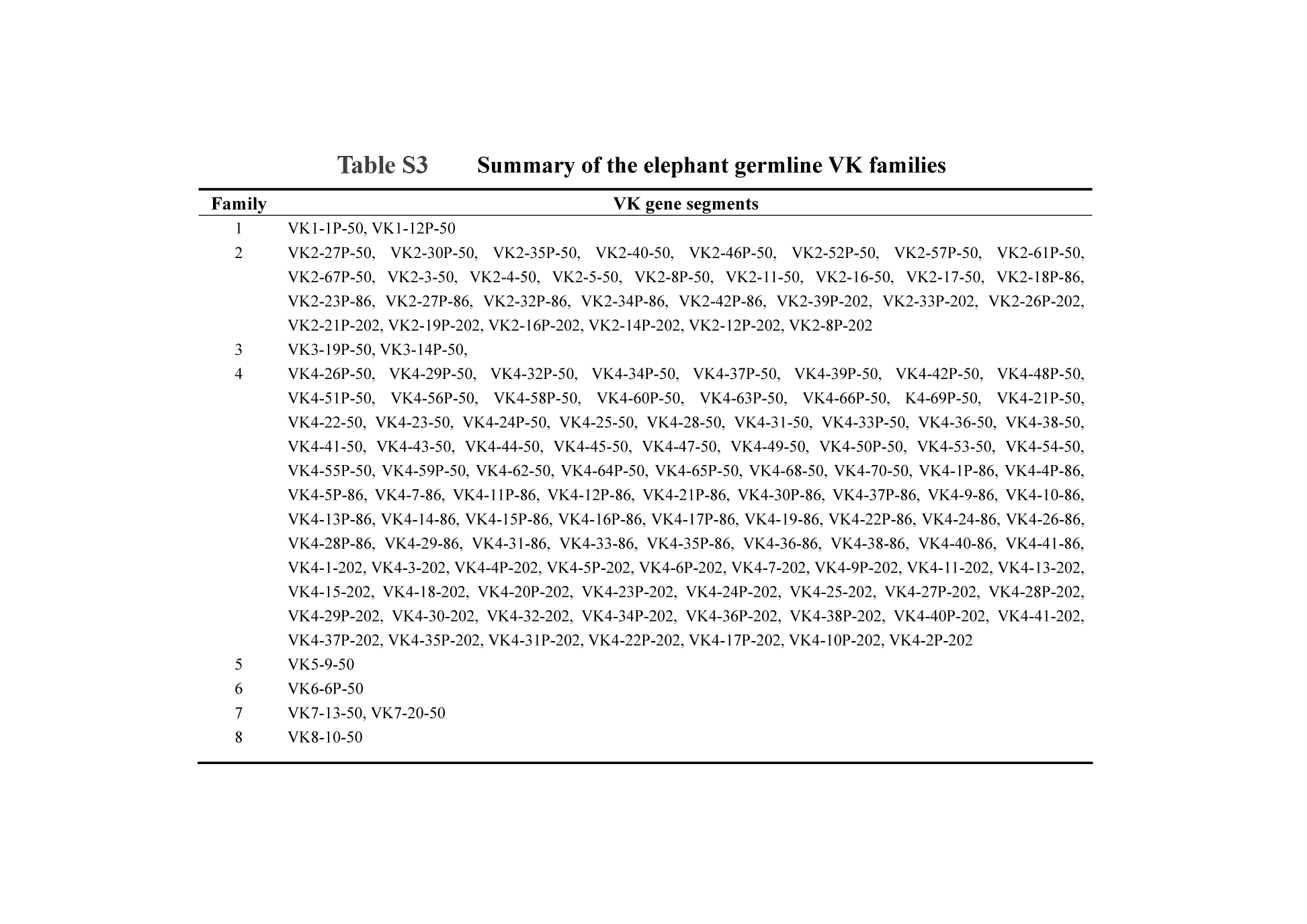

Supplement: Table S3 — The eight elephant Vκ gene families from scaffolds 202, 50, and 86. (TIF) [file pone.0016889.s008.tif]
